# Barriers to and facilitators of employment for people with psychiatric disabilities in Africa: a scoping review

**DOI:** 10.1080/16549716.2018.1463658

**Published:** 2018-05-15

**Authors:** I. D. Ebuenyi, E. V. Syurina, J. F. G. Bunders, B. J. Regeer

**Affiliations:** Athena Institute, Amsterdam Public Health Research Institute, Vrije Universiteit Amsterdam, Amsterdam, The Netherlands

**Keywords:** Psychiatric disability, social stigma, personal decision, supported employment, low- and middle-income countries

## Abstract

**Background**: Despite the importance of inclusive employment, described in Goal 8 of the Sustainable Development Goals (SDGs), employment of persons with psychiatric disabilities in Africa is lower than among the general population.

**Objective**: The aim of this scoping review is to explore evidence related to the barriers to and facilitators of employment of persons with psychiatric disabilities in Africa.

**Methods**: A literature search was conducted using six relevant electronic databases of articles published between 1990 and 2017.

**Results**: Eight studies were identified and analysed regarding barriers and facilitators of employment of persons with psychiatric disabilities. The dynamic adaptation of the bio-psycho-social model was used as an analytical framework. Identified barriers include ill health, (anticipated) psychiatric illness, social stigma and discrimination, negative attitudes among employers and the lack of social support and government welfare. Facilitators of employment include stability of mental illness, heightened self-esteem, a personal decision to work despite stigma, competitive and supported employment, reduction in social barriers/stigma and workplace accommodations.

**Conclusion**: Employment of persons with psychiatric disabilities is essential, yet there is dearth of scientific evidence to identify contextual models that might be useful in African countries and other low-and middle countries (LMICs). This gap in information would benefit from further research to improve the employment rates of persons with psychiatric disabilities in Africa.

## Background

Worldwide, employment rates among people with psychiatric disability are significantly lower than those of general population and even of individuals with other types of disabilities [1,2]. According to the Organization for Economic Co-operation and Development (OECD), mental health problems constitute 30–45% of all disability claims [3]. Some studies report that the employment rate among this group is 40% lower, while others state that only 25% of those with a mental disability are employed [3]. Studies suggest that this employment gap is especially evident in low- and middle-income countries (LMICs) because of the underlying socioeconomic and political reasons affecting the employment market and social welfare policies [1,2].

**Figure 1.**
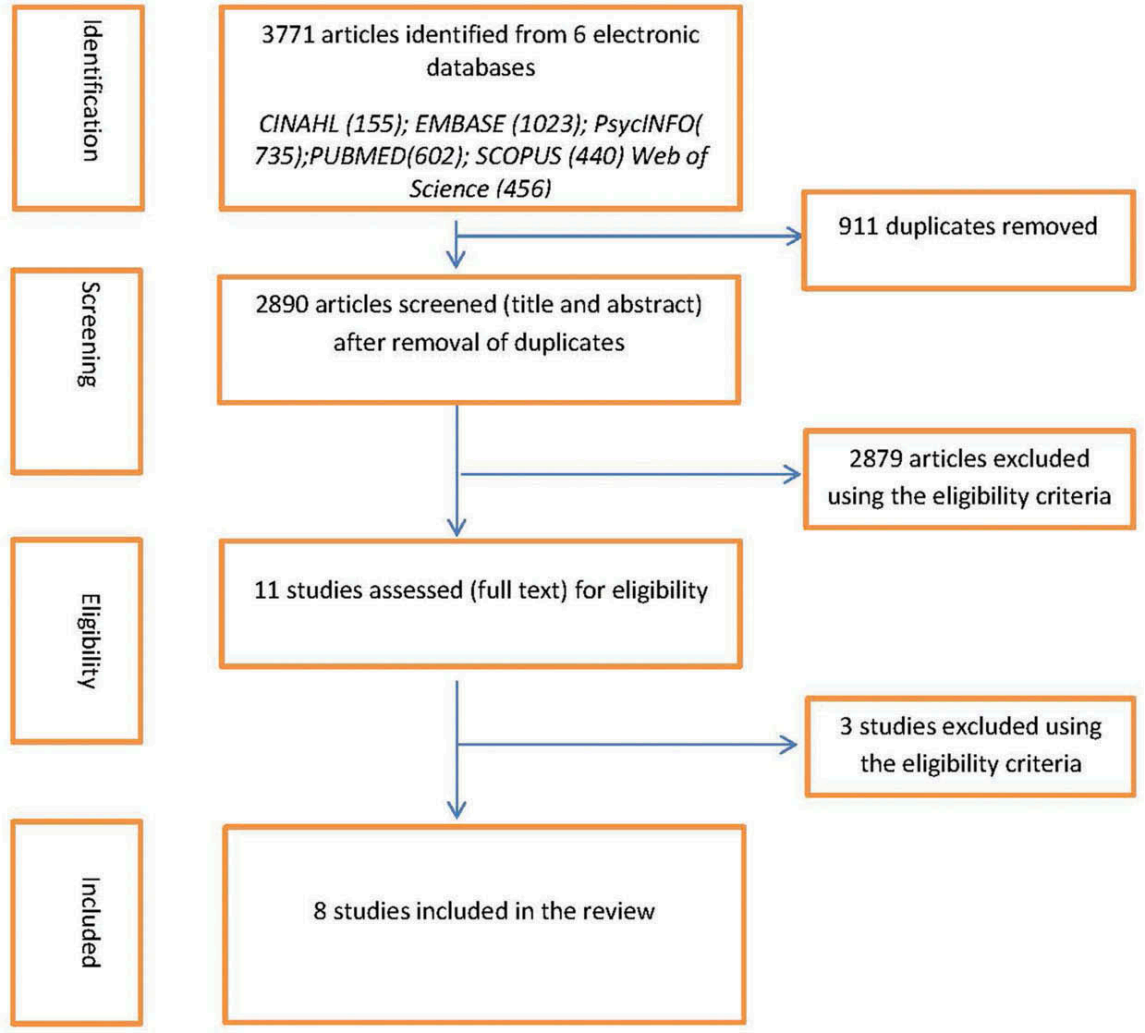
PRISMA flowchart of study selection process.

**Figure 2.**
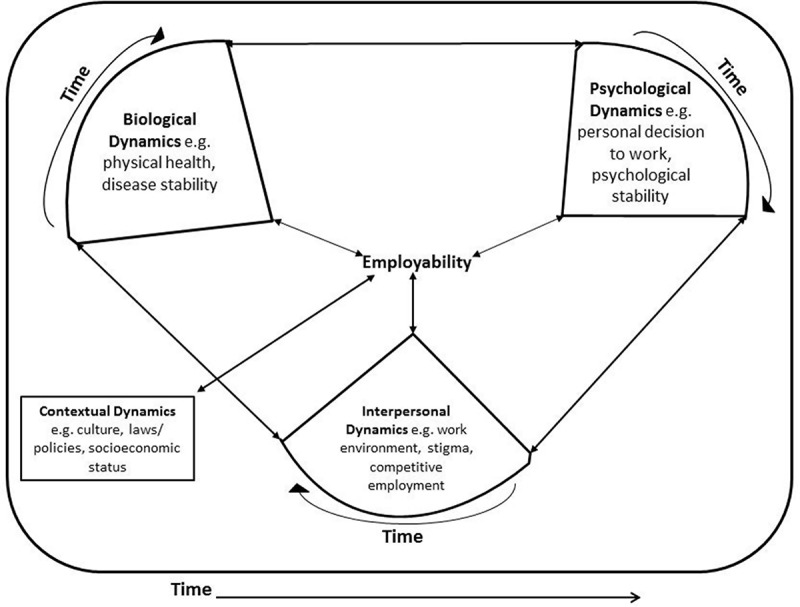
Adapted from Lehman et al. [25] showing the dynamic interaction of bio-psycho-social factors in the employability of persons with psychiatric disabilities.

Such an employment gap between people with a mental disability and the general population can only partially be explained by the disorder; true employment rates may be masked by stigma and discrimination that are closely associated with mental health issues [4]. These often militate against programmes that aim to improve the employability of persons with a disability [2,5]. Such misconceptions have huge ethical implications and socioeconomic effects on the lives of those affected [2], as it is known that increased economic participation is not only financially advantageous but also has a positive impact on the course of disease [6] and prevents recurrence [7].

When discussing mental health-related disabilities, it is important to highlight the lack of consensus on what constitutes mental disability. Severe mental illnesses such as schizophrenia, bipolar disorder and schizoaffective disorders are major causes of mental disability [8]. Also, globally, depression is one of the leading causes of disability [9], but in some African countries it is hard if not impossible to get a disability status approved for Common Mental Disorders (CMD) [10]. Taking this contextual specificity in mind, this review focuses on severe mental health disorders.

In order to be able to influence the employment rates of people with mental health disabilities, it is important to have an overview of existing barriers and known facilitators. Among the known barriers are: illness-specific factors, discriminatory attitudes among employers, lack of education and skills, and the failure to implement government provisions and recommendations for employment of persons with disabilities [11]. To overcome such barriers, studies recommend models such as supported employment (SE) for the employment of persons with mental disability [12]. In some settings, microfinance and cash transfers have been recommended to boost self-employment as an alternative to SE programmes [2,13], especially in LMICs.

Although there are evidence-based studies on barriers to and facilitators of employment of persons with a mental disability in HICs [5,12,14], few studies have systematically explored the subject in African countries [15]. This, combined with calls in the United Nations Convention on the Rights of Persons with Disabilities (UNCRPD) [16] and the Sustainable Development Goals (SDGs) [17], have advocated concerted action to protect the rights of persons with disabilities and their empowerment through inclusion in socioeconomic programmes. In this vein, this study adopts the exploratory approach of scoping reviews [18] and evaluates qualitative and quantitative studies on psychiatric disability in Africa in order to identify the barriers to and facilitators of employability for individuals with a psychiatric disability as well as existing employment models for persons with psychiatric disabilities in the region. In this study, psychiatric and mental disability are used interchangeably and refer to individuals with a form of mental illness that affects their social and occupational functioning [19].

## Theoretical approach

The issue of employment of individuals with a psychiatric disability can be influenced by a wide range of factors, of which some are linked to the individual in question, namely sex [20], age, specific diagnosis, duration and severity of the condition [21,22]; while others are more linked to the surrounding environment, i.e. family structure and support [23], prevalence of stigmatizing beliefs in the community and existing policy documents [24]. In order to discuss this broad spectrum in a systematic way, the expansion of the bio-psycho-social model by [25] was adopted. This model builds on conventional bio-psycho-social approaches [26] by introducing a dynamic systems perspective and applying Bronfenbrenner’s theories of development in order to underline the social influences.

Beyond the original three elements – biological (physical elements and body characteristics), psychological (cognitive, emotional, motivational, attitudinal, and behavioural system) and interpersonal (effects of actual or perceived social contacts on micro, meso and exo-levels) – the model adds the contextual aspects (broad-range culture, norms, policies, and values) and specifically focuses on the way the four groups influence each other and the person’s health. Moreover, it takes a developmental perspective on the elements, taking into account that they change over time, and hence are referred to as ‘dynamics’.

## Methods

### Search strategy

The review was conducted using the Joanna Briggs Institute (JBI) Methodology for Scoping Reviews [18]. The objectives, inclusion criteria and methods were specified in advance in the study protocol.

#### Data collection

A systematic search was undertaken across six relevant databases (CINAHL, EMBASE, PsycINFO, PUBMED, SCOPUS and Web of Science) on 9 March 2017. An updated search was conducted in September 2017. The search terms were based on the synonyms of: mental/psychiatric disabilities; employment; and a list of all African countries. Boolean operators AND; OR and NOT were used to construct the syntax (see Appendix). All relevant articles relating to the employment of people with a psychiatric disability in Africa from 1990 to September 2017 were captured. Identified articles were exported to Endnote.

The initial search yielded 3771 papers and this was reduced to 2890 after eliminating 911 duplicates. Title and abstract screening was conducted using the predefined eligibility criteria (Table 1) by IDE and EVS and 11 articles were selected for full-text screening, following which eight were selected for the review. The process of study selection, performed according to the Preferred Reporting Items for Systematic Reviews and Meta-Analyses (PRISMA) [27], can be found in Figure 1.

**Table 1.** Eligibility criteria.

	Inclusion	Exclusion
Study Design	Quantitative and qualitative studies including RCT, non-randomized controlled trials, cross-sectional studies, cohort studies and case-control.	Case reports/series, editorials, opinion pieces, interviews, systematic reviews, or books.
Date of Publication	None	None
Language	All Languages	None
Study Population	All adults 18 years or above diagnosed with mental/psychiatric disability	Adolescents/children under 18 years, diagnosis of Common Mental Disorder only
Study Outcome	Barriers (illness, stigma/discrimination, lack of skills/education, absence of legislation and government support) and opportunities (social support, education, information, government support and policies, employment/rehabilitation models for employment) for study subjects	Other outcomes unrelated to outcome of interest

#### Analysis

To synthesize the collected evidence, an extraction table was created. The following items were included: author, year of publication, study setting (including country), study design, study population, sample description and setting, main findings and limitations. The extraction was conducted by IDE and reviewed together with EVS BJR and JFGB. A summary of the data extraction is presented in Table 2.

**Table 2.** Study characteristics and findings.

Author & Year	Country	Study Design/Methods	Study Population/Setting	Study Aim	Main findings	Limitation
Oyefeso, 1994 (30)	Nigeria	Cross-sectional survey using self-administered questionnaires (attitude towards the work behaviour of ex-mental patients scale (ATWBS)	480 senior federal civil servants from 17 federal ministries in Nigeria	To examine the attitude of senior civil servants towards the work behaviour of ex-mental patients	77% of respondents had never worked with ex-mental patients and 72% indicated their unwillingness to work with them. 67% said the government was not doing enough to protect the rights of ex-mental patients while they are hospitalized. Only 12% are aware of welfare policies for ex-mental patients in the workplace and 88% agreed that there is need for a welfare policy for ex-mental patients in the workplace.	The response rate of sample of population was 76%.
Herzig and Thole 1998 (34)	Malawi	Cross-sectional survey	58 employers from Mzuzu, Malawi	To explore how the attitude of local employers affects employment of people with mental illness	52% of employers stated their willingness to employ psychiatric patients if they were currently well and recovered; and were willing to provide workplace accommodations for them. In a comparison between asthma and schizophrenia, 28% (13) of employers said they would hire neither; 11%(5) preferred to employ the patient with asthma while 9% (4) (employers needing labourers) would rather hire the psychiatric patient. All but one of the employers stated there was no incentive for them to hire persons with disability or illness.	The response rate of sample of population was 76%. The authors noted that the employers may have offered socially desirable answers.
Eaton, 2008 (29)	Nigeria	Cross-sectional survey using structured questionnaire.	41 ex-residents (severe mental illness) of Amaudo Itumbauzo (South East Nigeria) vocational rehabilitation programme.	To examine work engagement of ex-residents discharged from the rehabilitation facility	18 (36%) were engaged in the work activity and out of these number,10 were farming,6 were trader and the remaining 2 were involved in hairdressing and manual labour. 82% of those working saw the community psychiatric nurse in the last month. Mental illness (27.5%), physical illness (12.8%), social factors (e.g. family rejection) (17.5%) and lack of tools/equipment are the reported barriers for not working. Among those not working, 52% never resumed work after discharge.	Interviewer administered questionnaires may lead to socially desirable answers.
Boyce et al., 2009 (35)	Ghana	Cross-sectional Survey	400 mentally ill people (clinical diagnosis) in Basics Needs (Ghana) development programme	To examine the relationship between socioeconomic indicators (e.g. asset ownership, job retention after mental illness) and improvement/stability of mental illness.	Stability of illness and high self-esteem are associated with employment and job retention. Onset of mental illness is associated with job loss (OR-4.86;1.51–15.63) (NB unstable-last experienced illness≤6 months ago; stable-≥7months ago)	Illness stability is subjective and may be affected by recall bias. Interviewer administered questionnaires may lead to socially desirable answers.
Niekerk, 2009 (31)	South Africa	Qualitative Interviews using interpretive biography	17 persons with psychiatric disability (axis 1 of DSM IV) from the Western Cape, South Africa	To examine the factors that affect the work lives of people with psychiatric disability	Stigma and discrimination affected work opportunities of persons with psychiatric disability. Persons with psychiatric disabilities internalize societal stereotypes that they cannot work, which reduces their work ability. Fluctuations in health (psychiatric illness) led to job loss. Resolving the identity crisis (work ability), flexibility/job change, self-esteem and a personal decision to work enhances work decision and employment. Competitive employment is very helpful for resolving identity crisis.	Purposive sampling was used.
Atilola et al., 2014 (28)	Nigeria	Cross-sectional survey using self-administered questionnaires and the modified version of Link’s Discrimination devaluation (LDD) scale	90 Human resource (HR) personnel and employers from companies in Lagos, Nigeria.	To examine the familiarity and attitude of Human resource personnel to mental health issues in workplace.	53.3% of respondents have negative attitude towards mental health issues in the workplace and 72.2% would rather employ someone with a physical disability than a mental illness. 73.3% would not want to share an office with someone with mental illness. Workplace safety (82.5%) was the major restraint in recommending for employment someone with mental illness followed by work productivity (68.6%), reaction of other employees (56.85%) and financial burden of possible care (36.8%)	The response rate of companies sampled was 18.4%
Niekerk, et al., 2015 (33)	South Africa	Longitudinal descriptive study	10 persons with psychiatric disability utilizing supported employment services from a psychiatric hospital in Cape Town, South Africa.	To describe the parts of SE services used by persons with mental disability	SE is a useful option for return to work for person with psychiatric disabilities. Permanent jobs obtained by persons with psychiatric disabilities include vegetable farming, paper making, food production and book packaging.	Purposive sampling was used.
Niekerk, 2016 (32)	South Africa	Qualitative Interviews using interpretive biography.	17 persons with psychiatric disability (axis 1 of DSM IV) from the Western Cape, South Africa.	To examine the concept of work identity in the workplace by persons with psychiatric disability	Coming to terms with mental illness and decision to fight the social factors enhances employment opportunities. Flexibility in self-identity enhanced worker role. Psychiatric illness led to disruption of work and fear/anticipation of future relapse affected employment.	Purposive sampling was used.

Once data extraction had been completed, a narrative synthesis [28] was undertaken, based on the study objective and the exploratory nature of the study. All the findings of each paper were analyzed and coded according to the divisions of the bio-psycho-social model; coding findings from each category were synthesized, analyzed and presented in a narrative way.

## Results

Eight studies were included in the analysis. We observed a dearth of studies on the barriers to and facilitators of employments of persons with psychiatric disabilities in African countries. The identified studies had a varied, yet relatively limited, geographical range: three each were from Nigeria [29–31] and South Africa [32–34] and the remaining two were from Malawi [35] and Ghana [36].

Methodologically, six of the studies were quantitative: five had a cross-sectional design [29–31,35,36], one was a longitudinal study [34], and two used a qualitative research approach [32,33].

Five of the studies focused on experiences of individuals diagnosed with psychiatric disorders [30,32–34,36], while the remaining three investigated the perspectives of human resource personnel [29], employers [35] and senior civil servants [31].

Two of the studies of persons with psychiatric disabilities were in settings offering vocational rehabilitation services [30,36], one was in a clinical setting offering SE [34] while the other two explored work-participation perceptions of individuals with psychiatric illness in society in general [32,33].

Identified barriers and facilitators will be discussed separately in accordance with the dynamic representation of bio-psycho-social model by Lehman et al. [25].

Our choice to contextualize the results using the bio-psychosocial model was to ensure a systematic presentation of all the different factors at play. Although, it would have been useful to present the results according to the different actors relevant to employability, the available data made this impossible. For instance, the studies on employers or individuals involved in employment decisions focused on the attitude of employers and not their perceptions on barriers and on how to improve employment for person with mental disabilities.

### Barriers of employment for people with psychiatric disability

Our analysis identified five major clusters of barriers to employment: presence and severity of illness, underlying psychological load (including fear of relapse), social stigma, discrimination and negative attitudes by others, lack of skills/education and absence of policy support.

#### Biological factors

One of the most frequently named barriers to successful employment for people with mental health disabilities was ill health. Some papers talked about the effects of the more physical symptoms [30]. From the sample of 41 ex-residents of a vocational rehabilitation programme, 12.8% named the presence of co-morbid physical illness as a barrier to obtaining work. Most of the other papers identified psychiatric illness itself as a limitation to employment and participation in employment or job-related activities [30,32,33,36]. Mental illness was the highest (27.5%) self-reported barrier to the inability to work in a study to explore the work engagement of former residents of a psychiatric rehabilitation centre [30]. In addition, the fluctuation of psychiatric illness was strongly associated with job loss [32,33,36] and affected the desire to seek employment in the studies of persons with psychiatric illness.

#### Psychological factors

Niekerk and colleagues showed that apart from the presence of the mental health disorder itself, anticipation and fear of a relapse in symptoms was associated with a reduced desire to seek or retain employment in persons with psychiatric disorders [33].

#### Interpersonal/social factors

The interpersonal factors that influence the ability and motivation to find employment can broadly be described as prevalence of negative and stigmatizing attitudes and beliefs. Social stigma and discrimination were reported as a barrier to employment for people with psychiatric disabilities in six of the eight studies included in the study [29–33,35]. Such beliefs can be held by different actors in the individual’s environment, including family members and employers or their representatives (e.g. human resource personnel and senior civil servants).

In a cross-sectional survey in Nigeria, Eaton [30] reported that family rejection (17.5%) of persons with severe mental illness was the second highest self-reported barrier to employment after psychiatric illness in former residents of a psychiatric rehabilitation centre . Further, self-stigma plays a role – it was identified that a prevalent social stereotype in South Africa is that people with psychiatric disabilities cannot work and affected individuals internalize these perceptions, which also reduces their ability to work or motivation to secure employment [32].

Several of the identified papers discussed the employers’ and their representatives’ beliefs and attitudes as employment barriers. A survey of human resource personnel in Nigeria revealed that 72.2% of respondents would rather work with people with physical than mental illness and 73.3% would not want to share an office with someone with a mental illness [29]. Workplace safety was the major reason the human resource personnel would not recommend someone with mental illness for employment, while the perception that people with mental illness would be a burden was the least concern [29].

Negative attitudes among employers observed in a survey of senior federal civil servants in Nigeria found that 72% of the respondents indicated their unwillingness to work with people with mental illness and 77% had never done so [31]. In the same study, 67% of respondents agreed that government welfare for the care of persons with mental illness was sub-optimal. In a cross-sectional survey in Malawi, Herzig and Thole [35] reported that although 52% of employers indicated their willingness to hire persons with a psychiatric illness, they would only do so if they were ‘currently stable. In the same study, employers were more likely to hire someone with asthma (11%) than schizophrenia (9%) while 28% declared they would hire neither. In two qualitative studies in South Africa, both experienced stigma and anticipated discrimination were noted barriers to the employment of people with psychiatric disabilities [32,33].

#### Contextual factors

Among the identified contextual factors were lack of tools for work and relative absence of government policy support.

The study by Eaton [30] showed that lack of farming tools or equipment was the highest (32.5%) self-reported barrier to the ability to work among people with severe mental illness. This was often because of lack of money to buy or replace spoilt tools or the inability of the vocational training centre to replace the tools. Refusal to work (2.5%) and forgotten skills (5%) were other self-reported barriers in persons with mental illness [30].

Linked to that, several studies discussed a more structural, higher-level barrier: lack of institutionalized policy protection for people with mental illnesses. In the study by Oyefeso et al. [31] it was reported that 67% of the participating senior civil servants think that the government was not doing enough to protect the mental health patients. Moreover, 88% of respondents agreed that there was a need for a welfare policy for people who had been mental health patients. In the study by Herzig and Thole [35], while 52% from the sample of 58 employers were willing to employ a person with mental health disability, all but one reported the absence of policy or other incentives for them to do so.

### Facilitators of employment for people with psychiatric disability

We found five clusters of facilitators of employment for person with psychiatric disabilities, namely stability or reduced severity of mental illness, resolution of psychological conflicts, workplace accommodations, reduction in social barriers/stigma and governmental support.

#### Biological factors

Stability of mental illness and utilization of mental health services were reported as facilitators of employment and job retention [30,36]. Job retention was two (OR = 2.19,95% CI = 1.27–3.78) and five times (OR = 4.86,95% CI = 1.51–15.63) more likely in those with stable and improved mental illness respectively [36]. The association between working and stable mental health care was noted in a cohort of individuals with severe mental illness, where 82% of those who were working sought care from the community psychiatric nurse (CPN) in the last month [30].

#### Psychological factors

Resolution of a personal psychological conflict, heightened self-esteem and a personal decision by an individual with a psychiatric disability to work in spite of stigma are reported as major determinants of employment and work [32–34]. In two separate qualitative studies with individuals with psychiatric disabilities, Niekerk [32,33] reported that the personal decision to work [32] and coming to terms with mental illness and consciously deciding to overcome the social barriers [33] enhances employment opportunities. Self-esteem and confidence in one’s work ability increased the likelihood of employment and job retention [32,33]. These observations by Niekerk [33] are captured in the statement:

A further trend was that participants who were more at ease with the effects of psychiatric impairment on their identity and functioning seemed better able to maintain their roles….. participants who resumed work despite the occurrence/continuation of mild symptoms were more successful in maintaining such participation [33].

#### Interpersonal

Among the interpersonal factors facilitating employment are the ones linked to the direct working environment of individuals with mental disabilities.

In her study, Niekerk [32] identified that competitive employment was helpful for resolving an identity crisis of individuals with a disability and thus facilitate their employment. In a later study by the same author, it was shown that SE and provision of additional assistance during work is useful for the return to work for people with mental disabilities [34].

#### Contextual factors

Reduction in social stigma and improved social welfare were also reported facilitators of employment for people with psychiatric disabilities [29,31–34]. The survey of senior federal civil servants in Nigeria reported that 88% of respondents agreed that welfare policy in the workplace is essential for the employment of a person with mental illness. In addition, workplace accommodations were identified as relevant for improved work opportunities for people with psychiatric disabilities [31].

Despite the fact that in this study we identified certain biological, psychological, interpersonal and contextual factors that are shown to influence the employment of our study group, it is also important to bear in mind the interconnectedness and mutual influence of these factors as presented by Lehman et al. [25]. All the biological, psychological and interpersonal challenges that persons with psychiatric disabilities experience are interrelated and also influenced by contextual factors that operate in their environment. These factors (inter)act to determine the specific barriers and facilitators to employability the individual experiences (Figure 2). They are in constant flux and the dynamic interaction between them determines whether an individual secures or retains employment amidst the challenges of physical and psychiatric illness, social support/network and the contextual factors in the local environment. For instance, an individual with co-morbid psychiatric illness and physical health challenges, who is also exposed to work-related stigma, may be able to stay in employment depending on the availability of a positive or negative social network. Another example could be an individual with a psychiatric illness who lives in a community where s/he is likely to be stigmatized, but who is protected by national law, has different employment possibilities than a person without such protection.

## Other facilitators of employability for people with psychiatric disabilities

Employment models such as sheltered or supported employment are used in HICs to facilitate the employment of persons with a disability [37–39]. Where they exist and are taken up, they increase the employment opportunities for persons with a disability [13,38]. In this study, we observed three models of employment for persons with psychiatric disabilities. First, self-employment through farming and other self-help schemes were identified as major means of employment. Of 18 individuals involved in work in a vocational rehabilitation programme for people with severe mental illness, 10 were involved in farming [30].

Second, cooperative income-generation groups of persons with psychiatric disabilities was reported as a useful model of employment and a pathway towards competitive employment and resolution of work identity crisis [32]. Cooperative income-generation groups were useful because the individuals were able to work among other disabled persons and so felt accepted [32].

Third, SE services which assist persons with psychiatric disabilities to engage in competitive employment were identified as a useful option for the return to work of people with psychiatric disabilities in resource-poor settings [34]. Where they exist, SE may serve to facilitate the employability of persons with psychiatric disabilities.

## Discussion

Our study observed a striking lack of evidence regarding the employment of people with psychiatric disability throughout Africa. Despite using broad selection criteria, we were able to identify just eight papers discussing the issue. There may be several explanations. In African countries, there is associated stigma and lack of interest in mental illness; a situation that is worsened by lack of human resources for mental health care [4,40]. The stigma may be related to the attribution of the etiology of mental illness to supernatural causes [41]. This understanding informed the proposal by Gureje and colleagues for a collaboration between traditional and complementary systems of medicine (TCM) and conventional biomedicine (CB) in the care of persons with mental illness [42]. In addition, there is a lack of interest in mental health and of the political will to develop policies to advance mental health care at the same pace as addressing other health challenges in Africa [43].

In this article, we presented an overview of the barriers to and facilitators of employment for people with psychiatric disabilities in Africa. The analysis was performed using the expansion of the bio-psycho-social model [25]. In the analysis, a certain imbalance of attention was noted, as there seems to be more research on barriers than on facilitators. This can, however, be explained by the importance of describing the field, before facilitating actions can be made. When analyzing the barriers to and facilitators for employment, it became visible that most can be seen as two sides of the same coin. Depending on the situation and approach they can either hinder work participation or facilitate employment.

The biological factors influencing work participation are linked to the physical or mental health of the individual in question. Several studies have noted that fluctuation in mental illness led to disruption of work and inability to continue in a job [30,32,33,36]. On the other hand, others have shown that stability in the course of mental illness can help individuals to find and maintain work [36]. Previous studies on severe mental illness found that untreated or current mental illness is associated with impaired social and occupational functioning [44]. One of the factors contributing to the stability of mental health issues is timely and adequate access to care: Eaton [30] also noted that among persons with mental illness who were in work, 82% had seen the community psychiatric nurse in the last month. Broader research shows that availability of treatment and its uptake are known facilitators of wellbeing and employment for people with psychiatric disabilities [45].

The biological barriers to employment seem to be closely linked to the psychological ones. It was shown that the anticipation of psychiatric illness affects work ability, because the individual feared that the illness may recur and lead to interrupted employment [32]. These findings are not unique to African countries, and have been observed in varied countries around the world [5,8,46]. Other studies also found that taking a personal decision to work and being able to establish a personal means of coping with the hardships of the disease and the associated stigma can have positive impact on work ability and retention [32–34]. This is useful because it highlights the importance of choice and yearning for survival in the face of overwhelming challenges. Every person’s ability to cope may differ, but its use in cognitive behavioural therapy (CBT) may be essential for health professionals who work with people with a psychiatric illness.

In order to foster these coping strategies, it is also important to address stigma and negative beliefs about the psychiatric disabilities, which several studies find constitutes a considerable interpersonal barrier to employment of our target group [32]. Pervasive and negative attitudes to the work ability of individuals with psychiatric illness were recorded in three studies conducted with human resource personnel [29], employers [35] and senior federal civil servants [31]. These findings are supported by several studies in HICs that have demonstrated that stigma and discrimination in both society and the workplace adversely affect the employment of persons with psychiatric illness [2,5,12,47]. It was also frequently noted that the reduction of social barriers and stigma is useful in the participation in work of people with psychiatric disabilities [30,32,33]. Krupa and colleagues recommend intervention strategies to reduce the harmful of effects of stigma at work [48]. These interventions would involve workplace accommodations and changes in pervasive attitudes and assumptions which were recorded as barriers to employment for people with mental disabilities in this study [29–33].

The identified contextual factors influencing the employment of people with psychiatric disabilities in Africa were connected both to more practical aspects (absence of necessary tools), and also highlighted a more structural problem – the absence of government policy support.

Limited access to the necessary tools and equipment for work was described as an underlying factor that could explain reduced work participation [30]. This finding highlights a close relationship between poverty and mental illness and the twin factors of social causation and social drift, closely associated with psychiatric disability [49]. This is especially instructive because in LMICS self-employment through farming and personal business provide employment for the majority and people with psychiatric disabilities who face social and institutional workplace exclusion [2]. It is also pertinent to state that the lack of finance may not be due to mental illness and there was no comparison group in the study to demonstrate an association between lack of farming tools and mental illness.

Last, but not the least, the need for government support for the issue of employment of people with psychiatric disabilities was highlighted in several papers [31,35] . There seems to be a notable lack of policy protection for the target group. At present, this notion is supported by the UNCRPD [16] and the SDGs [17]. Governments everywhere have a duty towards persons with disabilities through legislation and provision of basic facilities, such as health care [4,16]. In addition, legislation against discrimination and job quotas for people with psychiatric disabilities are suggested means by which governments can assist persons with psychiatric disabilities [2].

However, when reviewing the results of this study one should be aware that the barriers and facilitators identified do not exist in isolation, but rather mutually influence each other. This presents both a challenge and an opportunity for potential interventions in the field. And we advocate more research about this interconnectedness.

This study also recorded three models or sources of employment for people with psychiatric disabilities, namely self-employment [30], cooperative groups [32] and supported employment [34]. Self-employment through farming was noted as a major form of employment for individuals with psychiatric illness [30]. This is supported by the literature and is particularly useful in LMICs where formal employment is relatively scarce [2]. However, finance may be a limitation to self-employment as shown in this study [30] and the work by Heymann and colleagues [2]. Cooperative income-generation groups were observed to facilitate employment because they allowed individuals to work without fear of discrimination among people who understand them [32]. This assessment is corroborated by the use of sheltered workshops [50] and employment farms [39], which have been found to be useful in vocational rehabilitation for people with mental illness. Supported employment that fosters competitive employment for people with psychiatric disabilities was also identified as a useful employment model in resource-poor settings [34]. Individual placement and support has been demonstrated to be very effective in HICs [12,51], but its applicability in African countries and other LMICs is still limited by finances and lack of government support [2], which has also been noted in HICS [13].

The results of this study need to be interpreted with caution, taking the methodological and contextual factors into account. First, the studies included in this study represent a very broad spectrum, while being quite limited in number. In addition to the specific limitations of the individual studies, the inclusion of studies from diverse populations presents particular challenges for interpretation of the findings. Also, our study criteria excluded grey literature which may have covered issues relating to employment that is often not considered in health care research.

There was also diversity in the terms used to describe psychiatric disability. The use of the terms like ex-mental patient [31] and ex-residents [30] tend to suggest something different, although the studies set out to describe persons with severe mental illness. The self-reported barriers to and facilitators of employment are subjective and may not reflect the actual situation. In addition, most of the studies did not compare the observed barriers to and facilitators of employment in persons with psychiatric disabilities to the general population.

It is pertinent once again to underline the paucity of original studies that explore barriers to and facilitators of employment for individuals with severe mental illness in Africa. The few studies drawn upon in this review demonstrate an urgent need for focused research in this area. Perhaps the stigma associated with mental illness extends to research in mental illness. This may be true in Africa, where stigma of mental illness is rife and extended to mental health care providers.

## Conclusion

The employment of people with psychiatric disabilities is essential and has both human rights justifications and socioeconomic benefits for those affected, society and governments. The dearth of context-relevant scientific evidence in Africa is of concern. There is a lack of evidence and the existing evidence is highly fragmented and outdated. This gap in information would benefit from further research on how to improve the employment rates among persons with psychiatric disabilities in Africa and the achievement of Goal 8 of the SDGs.

## Paper context

Employment of persons with psychiatric disabilities is low in Africa. This paper reviews barriers to and facilitators of employment for such individuals in the region. Apart from overall scarcity of data, the use of bio-psycho-social-dynamic model highlights inequality in coverage in research. More attention is given to barriers, especially to stigma. Present analysis can serve as a roadmap, showing existing gaps, which in turn can be used as directions for future research.

## Appendix. Search Strategy

### PubMed Session Results (9 March 2017)

**Table:**

Search	Query	Items found
#4	**#1 AND #2 AND #3**	662
#3	**‘Absenteeism’[Mesh] OR ‘Convalescence’[Mesh] OR ‘Recovery of Function’[Mesh] OR ‘Sick Leave’[Mesh] OR ‘Disability Evaluation’[Mesh] OR ‘Work Capacity Evaluation’[Mesh] OR ‘Rehabilitation, Vocational’[Mesh] OR ‘Sickness Impact Profile’[Mesh] OR ‘Occupational Health’[Mesh] OR ‘return to work’[tiab] OR (evaluation*[tiab] AND (disability[tiab] OR work capacity[tiab])) OR ‘work disability’[tiab] OR ‘work incapacity’[tiab] OR ‘work incapability’[tiab] OR ‘work inhibition’[tiab] OR ‘working incapacity’[tiab] OR ‘medical leave’[tiab] OR ‘sick leave’[tiab] OR ‘disability leave’[tiab] OR absente*[tiab] OR ‘work absence’[tiab] OR ‘disability absence’[tiab] OR convalescen*[tiab] OR sick day*[tiab] OR illness day*[tiab] OR ‘recovery of function’[tiab] OR ‘functional recovery’[tiab] OR (recovery[ti] AND function*[ti]) OR ‘reintegration’[tiab] OR ‘reemployment’[tiab] OR ‘job reentry’[tiab] OR ‘presenteeism’[tiab] OR ‘sickness absence’[tiab] OR ‘work absenteeism’[tiab] OR ‘work day loss’[tiab] OR ‘work time loss’[tiab] OR ‘work productivity’[tiab] OR work function*[tiab] OR ‘work participation’[tiab] OR ‘work performance’[tiab] OR ‘performance at work’[tiab] OR ‘employment status’[tiab] OR ‘work status’[tiab] OR ‘occupational health’[tiab] OR ‘Sheltered Workshops’[Mesh] OR ‘Employment’[Mesh] OR ‘Work’[Mesh] OR employment[tiab] OR labor[tiab] OR work[tiab] OR working[tiab] OR workplace*[tiab] OR occupation*[tiab] OR vocation*[tiab] OR sheltered work*[tiab]**	1,285,806
#2	**‘Mentally Ill Persons’[Mesh] OR mentally ill[tiab] OR ‘Mental Disorders’[Mesh:NoExp] OR severe mental[tiab] OR ‘Psychotic Disorders’[Mesh] OR psychoses[tiab] OR psychosis[tiab] OR psychotic[tiab] OR ‘Schizophrenia’[Mesh] OR schizo*[tiab] OR ‘Bipolar Disorder’[Mesh] OR bipolar[tiab] OR psychiatric disabilit*[tiab] OR psychosocial disabilit*[tiab] OR psycho-social disabilit*[tiab] OR psychiatric disable*[tiab] OR psychosocial disable*[tiab] OR ‘Depressive Disorder, Major’[Mesh] OR major depress*[tiab]**	408,450
#1	**‘Africa’[Mesh] OR Africa*[tw] OR Algeria[tw] OR Angola[tw] OR Benin[tw] OR Botswana[tw] OR Burkina Faso[tw] OR Burundi[tw] OR Cameroon[tw] OR Cape Verde[tw] OR Central African Republic[tw] OR Chad[tw] OR Comoros[tw] OR Congo[tw] OR Cote d’Ivoire[tw] OR Djibouti[tw] OR Egypt[tw] OR Equatorial Guinea[tw] OR Eritrea[tw] OR Ethiopia[tw] OR Gabon[tw] OR Gambia[tw] OR Ghana[tw] OR Guinea[tw] OR Kenya[tw] OR Lesotho[tw] OR Liberia[tw] OR Libya[tw] OR Madagascar[tw] OR Malawi[tw] OR Mali[tw] OR Mauritania[tw] OR Mauritius[tw] OR Mayotte[tw] OR Morocco[tw] OR Mozambique[tw] OR Namibia[tw] OR Niger[tw] OR Nigeria[tw] OR Reunion[tw] OR Rwanda[tw] OR Senegal[tw] OR Seychelles[tw] OR Sierra Leone[tw] OR Somalia[tw] OR South Sudan[tw] OR Sudan[tw] OR Swaziland[tw] OR Tanzania[tw] OR Togo[tw] OR Tunisia[tw] OR Uganda[tw] OR Western Sahara[tw] OR Zambia[tw] OR Zimbabwe[tw]**	571,678

### 662 items

(‘Africa’[Mesh] OR Africa*[tw] OR Algeria[tw] OR Angola[tw] OR Benin[tw] OR Botswana[tw] OR Burkina Faso[tw] OR Burundi[tw] OR Cameroon[tw] OR Cape Verde[tw] OR Central African Republic[tw] OR Chad[tw] OR Comoros[tw] OR Congo[tw] OR Cote d’Ivoire[tw] OR Djibouti[tw] OR Egypt[tw] OR Equatorial Guinea[tw] OR Eritrea[tw] OR Ethiopia[tw] OR Gabon[tw] OR Gambia[tw] OR Ghana[tw] OR Guinea[tw] OR Kenya[tw] OR Lesotho[tw] OR Liberia[tw] OR Libya[tw] OR Madagascar[tw] OR Malawi[tw] OR Mali[tw] OR Mauritania[tw] OR Mauritius[tw] OR Mayotte[tw] OR Morocco[tw] OR Mozambique[tw] OR Namibia[tw] OR Niger[tw] OR Nigeria[tw] OR Reunion[tw] OR Rwanda[tw] OR Senegal[tw] OR Seychelles[tw] OR Sierra Leone[tw] OR Somalia[tw] OR South Sudan[tw] OR Sudan[tw] OR Swaziland[tw] OR Tanzania[tw] OR Togo[tw] OR Tunisia[tw] OR Uganda[tw] OR Western Sahara[tw] OR Zambia[tw] OR Zimbabwe[tw]) AND (‘Mentally Ill Persons’[Mesh] OR mentally ill[tiab] OR ‘Mental Disorders’[Mesh:noexp] OR severe mental[tiab] OR ‘Psychotic Disorders’[Mesh] OR psychoses[tiab] OR psychosis[tiab] OR psychotic[tiab] OR ‘Schizophrenia’[Mesh] OR schizo*[tiab] OR ‘Bipolar Disorder’[Mesh] OR bipolar[tiab] OR psychiatric disabilit*[tiab] OR psychosocial disabilit*[tiab] OR psycho-social disabilit*[tiab] OR psychiatric disable*[tiab] OR psychosocial disable*[tiab] OR ‘Depressive Disorder, Major’[Mesh] OR major depress*[tiab]) AND (‘Absenteeism’[Mesh] OR ‘Convalescence’[Mesh] OR ‘Recovery of Function’[Mesh] OR ‘Sick Leave’[Mesh] OR ‘Disability Evaluation’[Mesh] OR ‘Work Capacity Evaluation’[Mesh] OR ‘Rehabilitation, Vocational’[Mesh] OR ‘Sickness Impact Profile’[Mesh] OR ‘Occupational Health’[Mesh] OR ‘return to work’[tiab] OR (evaluation*[tiab] AND (disability[tiab] OR work capacity[tiab])) OR ‘work disability’[tiab] OR ‘work incapacity’[tiab] OR ‘work incapability’[tiab] OR ‘work inhibition’[tiab] OR ‘working incapacity’[tiab] OR ‘medical leave’[tiab] OR ‘sick leave’[tiab] OR ‘disability leave’[tiab] OR absente*[tiab] OR ‘work absence’[tiab] OR ‘disability absence’[tiab] OR convalescen*[tiab] OR sick day*[tiab] OR illness day*[tiab] OR ‘recovery of function’[tiab] OR ‘functional recovery’[tiab] OR (recovery[ti] AND function*[ti]) OR ‘reintegration’[tiab] OR ‘reemployment’[tiab] OR ‘job reentry’[tiab] OR ‘presenteeism’[tiab] OR ‘sickness absence’[tiab] OR ‘work absenteeism’[tiab] OR ‘work day loss’[tiab] OR ‘work time loss’[tiab] OR ‘work productivity’[tiab] OR work function*[tiab] OR ‘work participation’[tiab] OR ‘work performance’[tiab] OR ‘performance at work’[tiab] OR ‘employment status’[tiab] OR ‘work status’[tiab] OR ‘occupational health’[tiab] OR ‘Sheltered Workshops’[Mesh] OR ‘Employment’[Mesh] OR ‘Work’[Mesh] OR employment[tiab] OR labor[tiab] OR work[tiab] OR working[tiab] OR workplace*[tiab] OR occupation*[tiab] OR vocation*[tiab] OR sheltered work*[tiab])

### Embase.com Session Results (9 March 2017)

**Table:**

Search	Query	Items found
#4	**#1 AND #2 AND #3**	1,023
#3	**‘work’/exp OR ‘convalescence’/exp OR ‘Sickness Impact Profile’/exp OR ‘occupational health’/exp OR ‘employment’/exp OR ‘return to work’:ab,ti OR (evaluation* AND (disability NEAR/3 ‘work capacity’):ab,ti) OR ‘work disability’:ab,ti OR ‘work incapacity’:ab,ti OR ‘work incapability’:ab,ti OR ‘work inhibition’:ab,ti OR ‘working incapacity’:ab,ti OR ‘medical leave’:ab,ti OR ‘sick leave’:ab,ti OR ‘disability leave’:ab,ti OR absente*:ab,ti OR ‘work absence’:ab,ti OR ‘disability absence’:ab,ti OR convalescen*:ab,ti OR ‘sick day*’:ab,ti OR ‘illness day*’:ab,ti OR ‘recovery of function’:ab,ti OR ‘functional recovery’:ab,ti OR (recovery:ti AND function*:ti) OR reintegration:ab,ti OR reemployment:ab,ti OR ‘job reentry’:ab,ti OR presenteeism:ab,ti OR ‘sickness absence’:ab,ti OR ‘work absenteeism’:ab,ti OR ‘work day loss’:ab,ti OR ‘work time loss’:ab,ti OR ‘work productivity’:ab,ti OR ‘work function*’:ab,ti OR ‘work participation’:ab,ti OR ‘work performance’:ab,ti OR ‘performance at work’:ab,ti OR ‘employment status’:ab,ti OR ‘work status’:ab,ti OR ‘occupational health’:ab,ti OR employment:ab,ti OR labor:ab,ti OR work:ab,ti OR working:ab,ti OR workplace*:ab,ti OR occupation*:ab,ti OR vocation*:ab,ti OR ‘sheltered work*’:ab,ti**	1,689,072
#2	**‘mental patient’/exp OR ‘mentally ill’:ab,ti OR ‘mental disease’/de OR ‘severe mental’:ab,ti OR ‘psychosis’/exp OR psychoses:ab,ti OR psychosis:ab,ti OR psychotic:ab,ti OR schizo*:ab,ti OR ‘bipolar disorder’/exp OR bipolar:ab,ti OR ‘psychiatric disabilit*’:ab,ti OR ‘psychosocial disabilit*’:ab,ti OR ‘psycho-social disabilit*’:ab,ti OR ‘psychiatric disable*’:ab,ti OR ‘psychosocial disable*’:ab,ti OR ‘major depression’/exp OR ‘major depress*’:ab,ti**	590,330
#1	**‘Africa’/exp OR Africa*:ab,ti OR Algeria:ab,ti OR Angola:ab,ti OR Benin:ab,ti OR Botswana:ab,ti OR ‘Burkina Faso’:ab,ti OR Burundi:ab,ti OR Cameroon:ab,ti OR ‘Cape Verde’:ab,ti OR ‘Central African Republic’:ab,ti OR Chad:ab,ti OR Comoros:ab,ti OR Congo:ab,ti OR ‘Cote d Ivoire’:ab,ti OR Djibouti:ab,ti OR Egypt:ab,ti OR ‘Equatorial Guinea’:ab,ti OR Eritrea:ab,ti OR Ethiopia:ab,ti OR Gabon:ab,ti OR Gambia:ab,ti OR Ghana:ab,ti OR Guinea:ab,ti OR Kenya:ab,ti OR Lesotho:ab,ti OR Liberia:ab,ti OR Libya:ab,ti OR Madagascar:ab,ti OR Malawi:ab,ti OR Mali:ab,ti OR Mauritania:ab,ti OR Mauritius:ab,ti OR Mayotte:ab,ti OR Morocco:ab,ti OR Mozambique:ab,ti OR Namibia:ab,ti OR Niger:ab,ti OR Nigeria:ab,ti OR Reunion:ab,ti OR Rwanda:ab,ti OR Senegal:ab,ti OR Seychelles:ab,ti OR ‘Sierra Leone’:ab,ti OR Somalia:ab,ti OR ‘South Sudan’:ab,ti OR Sudan:ab,ti OR Swaziland:ab,ti OR Tanzania:ab,ti OR Togo:ab,ti OR Tunisia:ab,ti OR Uganda:ab,ti OR ‘Western Sahara’:ab,ti OR Zambia:ab,ti OR Zimbabwe:ab,ti**	550,791

### 1023 items

(‘africa’/exp OR africa*:ab,ti OR algeria:ab,ti OR angola:ab,ti OR benin:ab,ti OR botswana:ab,ti OR ‘burkina faso’:ab,ti OR burundi:ab,ti OR cameroon:ab,ti OR ‘cape verde’:ab,ti OR ‘central african republic’:ab,ti OR chad:ab,ti OR comoros:ab,ti OR congo:ab,ti OR ‘cote d ivoire’:ab,ti OR djibouti:ab,ti OR egypt:ab,ti OR ‘equatorial guinea’:ab,ti OR eritrea:ab,ti OR ethiopia:ab,ti OR gabon:ab,ti OR gambia:ab,ti OR ghana:ab,ti OR guinea:ab,ti OR kenya:ab,ti OR lesotho:ab,ti OR liberia:ab,ti OR libya:ab,ti OR madagascar:ab,ti OR malawi:ab,ti OR mali:ab,ti OR mauritania:ab,ti OR mauritius:ab,ti OR mayotte:ab,ti OR morocco:ab,ti OR mozambique:ab,ti OR namibia:ab,ti OR niger:ab,ti OR nigeria:ab,ti OR reunion:ab,ti OR rwanda:ab,ti OR senegal:ab,ti OR seychelles:ab,ti OR ‘sierra leone’:ab,ti OR somalia:ab,ti OR ‘south sudan’:ab,ti OR sudan:ab,ti OR swaziland:ab,ti OR tanzania:ab,ti OR togo:ab,ti OR tunisia:ab,ti OR uganda:ab,ti OR ‘western sahara’:ab,ti OR zambia:ab,ti OR zimbabwe:ab,ti) AND (‘mental patient’/exp OR ‘mentally ill’:ab,ti OR ‘mental disease’/de OR ‘severe mental’:ab,ti OR ‘psychosis’/exp OR psychoses:ab,ti OR psychosis:ab,ti OR psychotic:ab,ti OR schizo*:ab,ti OR ‘bipolar disorder’/exp OR bipolar:ab,ti OR ‘psychiatric disabilit*’:ab,ti OR ‘psychosocial disabilit*’:ab,ti OR ‘psycho-social disabilit*’:ab,ti OR ‘psychiatric disable*’:ab,ti OR ‘psychosocial disable*’:ab,ti OR ‘major depression’/exp OR ‘major depress*’:ab,ti) AND (‘work’/exp OR ‘convalescence’/exp OR ‘sickness impact profile’/exp OR ‘occupational health’/exp OR ‘employment’/exp OR ‘return to work’:ab,ti OR (evaluation* AND (disability NEAR/3 ‘work capacity’):ab,ti) OR ‘work disability’:ab,ti OR ‘work incapacity’:ab,ti OR ‘work incapability’:ab,ti OR ‘work inhibition’:ab,ti OR ‘working incapacity’:ab,ti OR ‘medical leave’:ab,ti OR ‘sick leave’:ab,ti OR ‘disability leave’:ab,ti OR absente*:ab,ti OR ‘work absence’:ab,ti OR ‘disability absence’:ab,ti OR convalescen*:ab,ti OR ‘sick day*’:ab,ti OR ‘illness day*’:ab,ti OR ‘recovery of function’:ab,ti OR ‘functional recovery’:ab,ti OR (recovery:ti AND function*:ti) OR reintegration:ab,ti OR reemployment:ab,ti OR ‘job reentry’:ab,ti OR presenteeism:ab,ti OR ‘sickness absence’:ab,ti OR ‘work absenteeism’:ab,ti OR ‘work day loss’:ab,ti OR ‘work time loss’:ab,ti OR ‘work productivity’:ab,ti OR ‘work function*’:ab,ti OR ‘work participation’:ab,ti OR ‘work performance’:ab,ti OR ‘performance at work’:ab,ti OR ‘employment status’:ab,ti OR ‘work status’:ab,ti OR ‘occupational health’:ab,ti OR employment:ab,ti OR labor:ab,ti OR work:ab,ti OR working:ab,ti OR workplace*:ab,ti OR occupation*:ab,ti OR vocation*:ab,ti OR ‘sheltered work*’:ab,ti)

### PsycINFO Session Results (9 March 2017)

**Table:**

Search	Query	Items found
#4	**#1 AND #2 AND #3**	735
#3	**DE ‘Employee Absenteeism’ OR DE ‘Recovery (Disorders)’ OR DE ‘Employee Leave Benefits’ OR DE ‘Disability Evaluation’ OR DE ‘Vocational Rehabilitation’ OR DE ‘Supported Employment’ OR DE ‘Vocational Evaluation’ OR DE ‘Work Adjustment Training’ OR DE ‘Reemployment’ OR DE ‘Occupational Health’ OR DE ‘Work-Related Illnesses’ OR DE ‘Sheltered Workshops’ OR DE ‘Employment Status’ OR DE ‘Job Performance’ OR DE ‘Employee Efficiency’ OR DE ‘Employee Productivity’ OR DE ‘Job Involvement’ OR DE ‘Work-Life Balance’ OR TI (‘return to work’ OR (evaluation* AND (disability OR ‘work capacity’)) OR ‘work disability’ OR ‘work incapacity’ OR ‘work incapability’ OR ‘work inhibition’ OR ‘working incapacity’ OR ‘medical leave’ OR ‘sick leave’ OR ‘disability leave’ OR absente* OR ‘work absence’ OR ‘disability absence’ OR convalescen* OR ‘sick day*’ OR ‘illness day*’ OR ‘recovery of function’ OR ‘functional recovery’ OR (recovery AND function*) OR reintegration OR reemployment OR ‘job reentry’ OR presenteeism OR ‘sickness absence’ OR ‘work absenteeism’ OR ‘work day loss’ OR ‘work time loss’ OR ‘work productivity’ OR ‘work function*’ OR ‘work participation’ OR ‘work performance’ OR ‘performance at work’ OR ‘employment status’ OR ‘work status’ OR ‘occupational health’ OR employment OR labor OR work OR working OR workplace* OR occupation* OR vocation* OR ‘sheltered work*’) OR AB (‘return to work’ OR (evaluation* AND (disability OR ‘work capacity’)) OR ‘work disability’ OR ‘work incapacity’ OR ‘work incapability’ OR ‘work inhibition’ OR ‘working incapacity’ OR ‘medical leave’ OR ‘sick leave’ OR ‘disability leave’ OR absente* OR ‘work absence’ OR ‘disability absence’ OR convalescen* OR ‘sick day*’ OR ‘illness day*’ OR ‘recovery of function’ OR ‘functional recovery’ OR reintegration OR reemployment OR ‘job reentry’ OR presenteeism OR ‘sickness absence’ OR ‘work absenteeism’ OR ‘work day loss’ OR ‘work time loss’ OR ‘work productivity’ OR ‘work function*’ OR ‘work participation’ OR ‘work performance’ OR ‘performance at work’ OR ‘employment status’ OR ‘work status’ OR ‘occupational health’ OR employment OR labor OR work OR working OR workplace* OR occupation* OR vocation* OR ‘sheltered work*’)**	675,680
#2	**DE ‘Mental Disorders’ OR DE ‘Psychosis’ OR DE ‘Affective Psychosis’ OR DE ‘Paranoia (Psychosis)’ OR DE ‘Reactive Psychosis’ OR DE ‘Schizophrenia’ OR DE ‘Acute Schizophrenia’ OR DE ‘Catatonic Schizophrenia’ OR DE ‘Paranoid Schizophrenia’ OR DE ‘Process Schizophrenia’ OR DE ‘Schizophrenia (Disorganized Type)’ OR DE ‘Schizophreniform Disorder’ OR DE ‘Undifferentiated Schizophrenia’ OR DE ‘Bipolar Disorder’ OR DE ‘Cyclothymic Personality’ OR DE ‘Major Depression’ OR DE ‘Anaclitic Depression’ OR DE ‘Dysthymic Disorder’ OR DE ‘Endogenous Depression’ OR DE ‘Late Life Depression’ OR DE ‘Postpartum Depression’ OR DE ‘Reactive Depression’ OR DE ‘Recurrent Depression’ OR DE ‘Treatment Resistant Depression’ OR TI (‘mentally ill’ OR ‘severe mental’ OR psychoses OR psychosis OR psychotic OR schizo* OR bipolar OR ‘psychiatric disabilit*’ OR ‘psychosocial disabilit*’ OR ‘psycho-social disabilit*’ OR ‘psychiatric disable*’ OR ‘psychosocial disable*’ OR ‘major depress*’) OR AB (‘mentally ill’ OR ‘severe mental’ OR psychoses OR psychosis OR psychotic OR schizo* OR bipolar OR ‘psychiatric disabilit*’ OR ‘psychosocial disabilit*’ OR ‘psycho-social disabilit*’ OR ‘psychiatric disable*’ OR ‘psychosocial disable*’ OR ‘major depress*’)**	386,976
#1	**TI (Africa* OR Algeria OR Angola OR Benin OR Botswana OR ‘Burkina Faso‘ OR Burundi OR Cameroon OR ‘Cape Verde‘ OR ‘Central African Republic‘ OR Chad OR Comoros OR Congo OR ‘Cote d’Ivoire‘ OR Djibouti OR Egypt OR ‘Equatorial Guinea’ OR Eritrea OR Ethiopia OR Gabon OR Gambia OR Ghana OR Guinea OR Kenya OR Lesotho OR Liberia OR Libya OR Madagascar OR Malawi OR Mali OR Mauritania OR Mauritius OR Mayotte OR Morocco OR Mozambique OR Namibia OR Niger OR Nigeria OR Reunion OR Rwanda OR Senegal OR Seychelles OR ‘Sierra Leone‘ OR Somalia OR ‘South Sudan’ OR Sudan OR Swaziland OR Tanzania OR Togo OR Tunisia OR Uganda OR ‘Western Sahara’ OR Zambia OR Zimbabwe) OR AB (Africa* OR Algeria OR Angola OR Benin OR Botswana OR ‘Burkina Faso‘ OR Burundi OR Cameroon OR ‘Cape Verde‘ OR ‘Central African Republic‘ OR Chad OR Comoros OR Congo OR ‘Cote d’Ivoire‘ OR Djibouti OR Egypt OR ‘Equatorial Guinea’ OR Eritrea OR Ethiopia OR Gabon OR Gambia OR Ghana OR Guinea OR Kenya OR Lesotho OR Liberia OR Libya OR Madagascar OR Malawi OR Mali OR Mauritania OR Mauritius OR Mayotte OR Morocco OR Mozambique OR Namibia OR Niger OR Nigeria OR Reunion OR Rwanda OR Senegal OR Seychelles OR ‘Sierra Leone‘ OR Somalia OR ‘South Sudan’ OR Sudan OR Swaziland OR Tanzania OR Togo OR Tunisia OR Uganda OR ‘Western Sahara’ OR Zambia OR Zimbabwe)**	88,673

### 735 items

(TI (Africa* OR Algeria OR Angola OR Benin OR Botswana OR ‘Burkina Faso’ OR Burundi OR Cameroon OR ‘Cape Verde’ OR ‘Central African Republic’ OR Chad OR Comoros OR Congo OR ‘Cote d’Ivoire’ OR Djibouti OR Egypt OR ‘Equatorial Guinea’ OR Eritrea OR Ethiopia OR Gabon OR Gambia OR Ghana OR Guinea OR Kenya OR Lesotho OR Liberia OR Libya OR Madagascar OR Malawi OR Mali OR Mauritania OR Mauritius OR Mayotte OR Morocco OR Mozambique OR Namibia OR Niger OR Nigeria OR Reunion OR Rwanda OR Senegal OR Seychelles OR ‘Sierra Leone’ OR Somalia OR ‘South Sudan’ OR Sudan OR Swaziland OR Tanzania OR Togo OR Tunisia OR Uganda OR ‘Western Sahara’ OR Zambia OR Zimbabwe) OR AB (Africa* OR Algeria OR Angola OR Benin OR Botswana OR ‘Burkina Faso’ OR Burundi OR Cameroon OR ‘Cape Verde’ OR ‘Central African Republic’ OR Chad OR Comoros OR Congo OR ‘Cote d’Ivoire’ OR Djibouti OR Egypt OR ‘Equatorial Guinea’ OR Eritrea OR Ethiopia OR Gabon OR Gambia OR Ghana OR Guinea OR Kenya OR Lesotho OR Liberia OR Libya OR Madagascar OR Malawi OR Mali OR Mauritania OR Mauritius OR Mayotte OR Morocco OR Mozambique OR Namibia OR Niger OR Nigeria OR Reunion OR Rwanda OR Senegal OR Seychelles OR ‘Sierra Leone’ OR Somalia OR ‘South Sudan’ OR Sudan OR Swaziland OR Tanzania OR Togo OR Tunisia OR Uganda OR ‘Western Sahara’ OR Zambia OR Zimbabwe)) AND (DE ‘Mental Disorders’ OR DE ‘Psychosis’ OR DE ‘Affective Psychosis’ OR DE ‘Paranoia (Psychosis)’ OR DE ‘Reactive Psychosis’ OR DE ‘Schizophrenia’ OR DE ‘Acute Schizophrenia’ OR DE ‘Catatonic Schizophrenia’ OR DE ‘Paranoid Schizophrenia’ OR DE ‘Process Schizophrenia’ OR DE ‘Schizophrenia (Disorganized Type)’ OR DE ‘Schizophreniform Disorder’ OR DE ‘Undifferentiated Schizophrenia’ OR DE ‘Bipolar Disorder’ OR DE ‘Cyclothymic Personality’ OR DE ‘Major Depression’ OR DE ‘Anaclitic Depression’ OR DE ‘Dysthymic Disorder’ OR DE ‘Endogenous Depression’ OR DE ‘Late Life Depression’ OR DE ‘Postpartum Depression’ OR DE ‘Reactive Depression’ OR DE ‘Recurrent Depression’ OR DE ‘Treatment Resistant Depression’ OR TI (‘mentally ill’ OR ‘severe mental’ OR psychoses OR psychosis OR psychotic OR schizo* OR bipolar OR ‘psychiatric disabilit*’ OR ‘psychosocial disabilit*’ OR ‘psycho-social disabilit*’ OR ‘psychiatric disable*’ OR ‘psychosocial disable*’ OR ‘major depress*’) OR AB (‘mentally ill’ OR ‘severe mental’ OR psychoses OR psychosis OR psychotic OR schizo* OR bipolar OR ‘psychiatric disabilit*’ OR ‘psychosocial disabilit*’ OR ‘psycho-social disabilit*’ OR ‘psychiatric disable*’ OR ‘psychosocial disable*’ OR ‘major depress*’)) AND (DE ‘Employee Absenteeism’ OR DE ‘Recovery (Disorders)’ OR DE ‘Employee Leave Benefits’ OR DE ‘Disability Evaluation’ OR DE ‘Vocational Rehabilitation’ OR DE ‘Supported Employment’ OR DE ‘Vocational Evaluation’ OR DE ‘Work Adjustment Training’ OR DE ‘Reemployment’ OR DE ‘Occupational Health’ OR DE ‘Work-Related Illnesses’ OR DE ‘Sheltered Workshops’ OR DE ‘Employment Status’ OR DE ‘Job Performance’ OR DE ‘Employee Efficiency’ OR DE ‘Employee Productivity’ OR DE ‘Job Involvement’ OR DE ‘Work-Life Balance’ OR TI (‘return to work’ OR (evaluation* AND (disability OR ‘work capacity’)) OR ‘work disability’ OR ‘work incapacity’ OR ‘work incapability’ OR ‘work inhibition’ OR ‘working incapacity’ OR ‘medical leave’ OR ‘sick leave’ OR ‘disability leave’ OR absente* OR ‘work absence’ OR ‘disability absence’ OR convalescen* OR ‘sick day*’ OR ‘illness day*’ OR ‘recovery of function’ OR ‘functional recovery’ OR (recovery AND function*) OR reintegration OR reemployment OR ‘job reentry’ OR presenteeism OR ‘sickness absence’ OR ‘work absenteeism’ OR ‘work day loss’ OR ‘work time loss’ OR ‘work productivity’ OR ‘work function*’ OR ‘work participation’ OR ‘work performance’ OR ‘performance at work’ OR ‘employment status’ OR ‘work status’ OR ‘occupational health’ OR employment OR labor OR work OR working OR workplace* OR occupation* OR vocation* OR ‘sheltered work*’) OR AB (‘return to work’ OR (evaluation* AND (disability OR ‘work capacity’)) OR ‘work disability’ OR ‘work incapacity’ OR ‘work incapability’ OR ‘work inhibition’ OR ‘working incapacity’ OR ‘medical leave’ OR ‘sick leave’ OR ‘disability leave’ OR absente* OR ‘work absence’ OR ‘disability absence’ OR convalescen* OR ‘sick day*’ OR ‘illness day*’ OR ‘recovery of function’ OR ‘functional recovery’ OR reintegration OR reemployment OR ‘job reentry’ OR presenteeism OR ‘sickness absence’ OR ‘work absenteeism’ OR ‘work day loss’ OR ‘work time loss’ OR ‘work productivity’ OR ‘work function*’ OR ‘work participation’ OR ‘work performance’ OR ‘performance at work’ OR ‘employment status’ OR ‘work status’ OR ‘occupational health’ OR employment OR labor OR work OR working OR workplace* OR occupation* OR vocation* OR ‘sheltered work*’) )

### Web of Science (Core Collection) Session Results (9 March 2017)

#### (Indexes = SCI-EXPANDED, SSCI, A&HCI, ESCI Timespan = All years)

**Table:**

Search	Query	Items found
#4	**#1 AND #2 AND #3**	456
#3	**TS = (‘return to work’ OR (evaluation* AND (disability OR ‘work capacity’)) OR ‘work disability’ OR ‘work incapacity’ OR ‘work incapability’ OR ‘work inhibition’ OR ‘working incapacity’ OR ‘medical leave’ OR ‘sick leave’ OR ‘disability leave’ OR absente* OR ‘work absence’ OR ‘disability absence’ OR convalescen* OR ‘sick day*’ OR ‘illness day*’ OR ‘recovery of function’ OR ‘functional recovery’ OR (recovery AND function*) OR reintegration OR reemployment OR ‘job reentry’ OR presenteeism OR ‘sickness absence’ OR ‘work absenteeism’ OR ‘work day loss’ OR ‘work time loss’ OR ‘work productivity’ OR ‘work function*’ OR ‘work participation’ OR ‘work performance’ OR ‘performance at work’ OR ‘employment status’ OR ‘work status’ OR ‘occupational health’ OR employment OR labor OR work OR working OR workplace* OR occupation* OR vocation* OR ‘sheltered work*’)**	2,625,491
#2	**TS = (‘mentally ill’ OR ‘severe mental’ OR psychoses OR psychosis OR psychotic OR schizo* OR bipolar OR ‘psychiatric disabilit*’ OR ‘psychosocial disabilit*’ OR ‘psycho-social disabilit*’ OR ‘psychiatric disable*’ OR ‘psychosocial disable*’ OR ‘major depress*’)**	371,923
#1	**TS = (Africa* OR Algeria OR Angola OR Benin OR Botswana OR ‘Burkina Faso’ OR Burundi OR Cameroon OR ‘Cape Verde’ OR ‘Central African Republic’ OR Chad OR Comoros OR Congo OR ‘Cote d’Ivoire’ OR Djibouti OR Egypt OR ‘Equatorial Guinea’ OR Eritrea OR Ethiopia OR Gabon OR Gambia OR Ghana OR Guinea OR Kenya OR Lesotho OR Liberia OR Libya OR Madagascar OR Malawi OR Mali OR Mauritania OR Mauritius OR Mayotte OR Morocco OR Mozambique OR Namibia OR Niger OR Nigeria OR Reunion OR Rwanda OR Senegal OR Seychelles OR ‘Sierra Leone’ OR Somalia OR ‘South Sudan’ OR Sudan OR Swaziland OR Tanzania OR Togo OR Tunisia OR Uganda OR ‘Western Sahara’ OR Zambia OR Zimbabwe)**	832,910

### 456 items

TS = (Africa* OR Algeria OR Angola OR Benin OR Botswana OR ‘Burkina Faso’ OR Burundi OR Cameroon OR ‘Cape Verde’ OR ‘Central African Republic’ OR Chad OR Comoros OR Congo OR ‘Cote d’Ivoire’ OR Djibouti OR Egypt OR ‘Equatorial Guinea’ OR Eritrea OR Ethiopia OR Gabon OR Gambia OR Ghana OR Guinea OR Kenya OR Lesotho OR Liberia OR Libya OR Madagascar OR Malawi OR Mali OR Mauritania OR Mauritius OR Mayotte OR Morocco OR Mozambique OR Namibia OR Niger OR Nigeria OR Reunion OR Rwanda OR Senegal OR Seychelles OR ‘Sierra Leone’ OR Somalia OR ‘South Sudan’ OR Sudan OR Swaziland OR Tanzania OR Togo OR Tunisia OR Uganda OR ‘Western Sahara’ OR Zambia OR Zimbabwe) AND TS = (‘mentally ill’ OR ‘severe mental’ OR psychoses OR psychosis OR psychotic OR schizo* OR bipolar OR ‘psychiatric disabilit*’ OR ‘psychosocial disabilit*’ OR ‘psycho-social disabilit*’ OR ‘psychiatric disable*’ OR ‘psychosocial disable*’ OR ‘major depress*’) AND TS = (‘return to work’ OR (evaluation* AND (disability OR ‘work capacity’)) OR ‘work disability’ OR ‘work incapacity’ OR ‘work incapability’ OR ‘work inhibition’ OR ‘working incapacity’ OR ‘medical leave’ OR ‘sick leave’ OR ‘disability leave’ OR absente* OR ‘work absence’ OR ‘disability absence’ OR convalescen* OR ‘sick day*’ OR ‘illness day*’ OR ‘recovery of function’ OR ‘functional recovery’ OR (recovery AND function*) OR reintegration OR reemployment OR ‘job reentry’ OR presenteeism OR ‘sickness absence’ OR ‘work absenteeism’ OR ‘work day loss’ OR ‘work time loss’ OR ‘work productivity’ OR ‘work function*’ OR ‘work participation’ OR ‘work performance’ OR ‘performance at work’ OR ‘employment status’ OR ‘work status’ OR ‘occupational health’ OR employment OR labor OR work OR working OR workplace* OR occupation* OR vocation* OR ‘sheltered work*’)

### Scopus Session Results (9 March 2017)

**Table:**

Search	Query	Items found
#4	**#1 AND #2 AND #3**	740
#3	**TITLE-ABS-KEY (‘return to work’ OR (evaluation* W/3 (disability OR ‘work capacity’)) OR ‘work disability’ OR ‘work incapacity’ OR ‘work incapability’ OR ‘work inhibition’ OR ‘working incapacity’ OR ‘medical leave’ OR ‘sick leave’ OR ‘disability leave’ OR absente* OR ‘work absence’ OR ‘disability absence’ OR convalescen* OR ‘sick day*’ OR ‘illness day*’ OR ‘recovery of function’ OR ‘functional recovery’ OR (recovery W/3 function*) OR reintegration OR reemployment OR ‘job reentry’ OR presenteeism OR ‘sickness absence’ OR ‘work absenteeism’ OR ‘work day loss’ OR ‘work time loss’ OR ‘work productivity’ OR ‘work function*’ OR ‘work participation’ OR ‘work performance’ OR ‘performance at work’ OR ‘employment status’ OR ‘work status’ OR ‘occupational health’ OR employment OR labor OR work OR working OR workplace* OR occupation* OR vocation* OR ‘sheltered work*’)**	5,013,307
#2	**TITLE-ABS-KEY (‘mentally ill’ OR ‘severe mental’ OR psychoses OR psychosis OR psychotic OR schizo* OR bipolar OR ‘psychiatric disabilit*’ OR ‘psychosocial disabilit*’ OR ‘psycho-social disabilit*’ OR ‘psychiatric disable*’ OR ‘psychosocial disable*’ OR ‘major depress*’)**	475,907
#1	**TITLE-ABS-KEY (Africa* OR Algeria OR Angola OR Benin OR Botswana OR ‘Burkina Faso‘ OR Burundi OR Cameroon OR ‘Cape Verde‘ OR ‘Central African Republic‘ OR Chad OR Comoros OR Congo OR ‘Cote d’Ivoire‘ OR Djibouti OR Egypt OR ‘Equatorial Guinea’ OR Eritrea OR Ethiopia OR Gabon OR Gambia OR Ghana OR Guinea OR Kenya OR Lesotho OR Liberia OR Libya OR Madagascar OR Malawi OR Mali OR Mauritania OR Mauritius OR Mayotte OR Morocco OR Mozambique OR Namibia OR Niger OR Nigeria OR Reunion OR Rwanda OR Senegal OR Seychelles OR ‘Sierra Leone‘ OR Somalia OR ‘South Sudan’ OR Sudan OR Swaziland OR Tanzania OR Togo OR Tunisia OR Uganda OR ‘Western Sahara’ OR Zambia OR Zimbabwe)**	1,124,759

### 740 items

TITLE-ABS-KEY (Africa* OR Algeria OR Angola OR Benin OR Botswana OR ‘Burkina Faso‘ OR Burundi OR Cameroon OR ‘Cape Verde‘ OR ‘Central African Republic‘ OR Chad OR Comoros OR Congo OR ‘Cote d’Ivoire‘ OR Djibouti OR Egypt OR ‘Equatorial Guinea’ OR Eritrea OR Ethiopia OR Gabon OR Gambia OR Ghana OR Guinea OR Kenya OR Lesotho OR Liberia OR Libya OR Madagascar OR Malawi OR Mali OR Mauritania OR Mauritius OR Mayotte OR Morocco OR Mozambique OR Namibia OR Niger OR Nigeria OR Reunion OR Rwanda OR Senegal OR Seychelles OR ‘Sierra Leone‘ OR Somalia OR ‘South Sudan’ OR Sudan OR Swaziland OR Tanzania OR Togo OR Tunisia OR Uganda OR ‘Western Sahara’ OR Zambia OR Zimbabwe) AND TITLE-ABS-KEY (‘mentally ill’ OR ‘severe mental’ OR psychoses OR psychosis OR psychotic OR schizo* OR bipolar OR ‘psychiatric disabilit*’ OR ‘psychosocial disabilit*’ OR ‘psycho-social disabilit*’ OR ‘psychiatric disable*’ OR ‘psychosocial disable*’ OR ‘major depress*’) AND TITLE-ABS-KEY (‘return to work’ OR (evaluation* W/3 (disability OR ‘work capacity’)) OR ‘work disability’ OR ‘work incapacity’ OR ‘work incapability’ OR ‘work inhibition’ OR ‘working incapacity’ OR ‘medical leave’ OR ‘sick leave’ OR ‘disability leave’ OR absente* OR ‘work absence’ OR ‘disability absence’ OR convalescen* OR ‘sick day*’ OR ‘illness day*’ OR ‘recovery of function’ OR ‘functional recovery’ OR (recovery W/3 function*) OR reintegration OR reemployment OR ‘job reentry’ OR presenteeism OR ‘sickness absence’ OR ‘work absenteeism’ OR ‘work day loss’ OR ‘work time loss’ OR ‘work productivity’ OR ‘work function*’ OR ‘work participation’ OR ‘work performance’ OR ‘performance at work’ OR ‘employment status’ OR ‘work status’ OR ‘occupational health’ OR employment OR labor OR work OR working OR workplace* OR occupation* OR vocation* OR ‘sheltered work*’)

### Cinahl Session Results (9 March 2017)

**Table:**

Search	Query	Items found
#4	**#1 AND #2 AND #3**	155
#3	**(MH ‘Absenteeism’) OR (MH ‘Recovery’) OR (MH ‘Sick Leave’) OR (MH ‘Disability Evaluation+’) OR (MH ‘Rehabilitation, Vocational+’) OR (MH ‘Sickness Impact Profile’) OR (MH ‘Occupational Health+’) OR (MH ‘Job Re-Entry’) OR TI (‘return to work’ OR (evaluation* AND (disability OR ‘work capacity’)) OR ‘work disability’ OR ‘work incapacity’ OR ‘work incapability’ OR ‘work inhibition’ OR ‘working incapacity’ OR ‘medical leave’ OR ‘sick leave’ OR ‘disability leave’ OR absente* OR ‘work absence’ OR ‘disability absence’ OR convalescen* OR ‘sick day*’ OR ‘illness day*’ OR ‘recovery of function’ OR ‘functional recovery’ OR (recovery AND function*) OR reintegration OR reemployment OR ‘job reentry’ OR presenteeism OR ‘sickness absence’ OR ‘work absenteeism’ OR ‘work day loss’ OR ‘work time loss’ OR ‘work productivity’ OR ‘work function*’ OR ‘work participation’ OR ‘work performance’ OR ‘performance at work’ OR ‘employment status’ OR ‘work status’ OR ‘occupational health’ OR employment OR labor OR work OR working OR workplace* OR occupation* OR vocation* OR ‘sheltered work*’) OR AB (‘return to work’ OR (evaluation* AND (disability OR ‘work capacity’)) OR ‘work disability’ OR ‘work incapacity’ OR ‘work incapability’ OR ‘work inhibition’ OR ‘working incapacity’ OR ‘medical leave’ OR ‘sick leave’ OR ‘disability leave’ OR absente* OR ‘work absence’ OR ‘disability absence’ OR convalescen* OR ‘sick day*’ OR ‘illness day*’ OR ‘recovery of function’ OR ‘functional recovery’ OR reintegration OR reemployment OR ‘job reentry’ OR presenteeism OR ‘sickness absence’ OR ‘work absenteeism’ OR ‘work day loss’ OR ‘work time loss’ OR ‘work productivity’ OR ‘work function*’ OR ‘work participation’ OR ‘work performance’ OR ‘performance at work’ OR ‘employment status’ OR ‘work status’ OR ‘occupational health’ OR employment OR labor OR work OR working OR workplace* OR occupation* OR vocation* OR ‘sheltered work*’)**	265,125
#2	**(MH ‘Mentally Disabled Persons’) OR (MH ‘Mental Disorders’) OR (MH ‘Psychotic Disorders+’) OR (MH ‘Schizophrenia+’) OR (MH ‘Bipolar Disorder+’) OR TI (‘mentally ill’ OR ‘severe mental’ OR psychoses OR psychosis OR psychotic OR schizo* OR bipolar OR ‘psychiatric disabilit*’ OR ‘psychosocial disabilit*’ OR ‘psycho-social disabilit*’ OR ‘psychiatric disable*’ OR ‘psychosocial disable*’ OR ‘major depress*’) OR AB (‘mentally ill’ OR ‘severe mental’ OR psychoses OR psychosis OR psychotic OR schizo* OR bipolar OR ‘psychiatric disabilit*’ OR ‘psychosocial disabilit*’ OR ‘psycho-social disabilit*’ OR ‘psychiatric disable*’ OR ‘psychosocial disable*’ OR ‘major depress*’)**	101,863
#1	**(MH ‘Africa+’) OR TI (Africa* OR Algeria OR Angola OR Benin OR Botswana OR ‘Burkina Faso‘ OR Burundi OR Cameroon OR ‘Cape Verde‘ OR ‘Central African Republic‘ OR Chad OR Comoros OR Congo OR ‘Cote d’Ivoire‘ OR Djibouti OR Egypt OR ‘Equatorial Guinea’ OR Eritrea OR Ethiopia OR Gabon OR Gambia OR Ghana OR Guinea OR Kenya OR Lesotho OR Liberia OR Libya OR Madagascar OR Malawi OR Mali OR Mauritania OR Mauritius OR Mayotte OR Morocco OR Mozambique OR Namibia OR Niger OR Nigeria OR Reunion OR Rwanda OR Senegal OR Seychelles OR ‘Sierra Leone‘ OR Somalia OR ‘South Sudan’ OR Sudan OR Swaziland OR Tanzania OR Togo OR Tunisia OR Uganda OR ‘Western Sahara’ OR Zambia OR Zimbabwe) OR AB (Africa* OR Algeria OR Angola OR Benin OR Botswana OR ‘Burkina Faso‘ OR Burundi OR Cameroon OR ‘Cape Verde‘ OR ‘Central African Republic‘ OR Chad OR Comoros OR Congo OR ‘Cote d’Ivoire‘ OR Djibouti OR Egypt OR ‘Equatorial Guinea’ OR Eritrea OR Ethiopia OR Gabon OR Gambia OR Ghana OR Guinea OR Kenya OR Lesotho OR Liberia OR Libya OR Madagascar OR Malawi OR Mali OR Mauritania OR Mauritius OR Mayotte OR Morocco OR Mozambique OR Namibia OR Niger OR Nigeria OR Reunion OR Rwanda OR Senegal OR Seychelles OR ‘Sierra Leone‘ OR Somalia OR ‘South Sudan’ OR Sudan OR Swaziland OR Tanzania OR Togo OR Tunisia OR Uganda OR ‘Western Sahara’ OR Zambia OR Zimbabwe)**	57,661

### 155 items

((MH ‘Africa+’) OR TI (Africa* OR Algeria OR Angola OR Benin OR Botswana OR ‘Burkina Faso’ OR Burundi OR Cameroon OR ‘Cape Verde’ OR ‘Central African Republic’ OR Chad OR Comoros OR Congo OR ‘Cote d’Ivoire’ OR Djibouti OR Egypt OR ‘Equatorial Guinea’ OR Eritrea OR Ethiopia OR Gabon OR Gambia OR Ghana OR Guinea OR Kenya OR Lesotho OR Liberia OR Libya OR Madagascar OR Malawi OR Mali OR Mauritania OR Mauritius OR Mayotte OR Morocco OR Mozambique OR Namibia OR Niger OR Nigeria OR Reunion OR Rwanda OR Senegal OR Seychelles OR ‘Sierra Leone’ OR Somalia OR ‘South Sudan’ OR Sudan OR Swaziland OR Tanzania OR Togo OR Tunisia OR Uganda OR ‘Western Sahara’ OR Zambia OR Zimbabwe) OR AB (Africa* OR Algeria OR Angola OR Benin OR Botswana OR ‘Burkina Faso’ OR Burundi OR Cameroon OR ‘Cape Verde’ OR ‘Central African Republic’ OR Chad OR Comoros OR Congo OR ‘Cote d’Ivoire’ OR Djibouti OR Egypt OR ‘Equatorial Guinea’ OR Eritrea OR Ethiopia OR Gabon OR Gambia OR Ghana OR Guinea OR Kenya OR Lesotho OR Liberia OR Libya OR Madagascar OR Malawi OR Mali OR Mauritania OR Mauritius OR Mayotte OR Morocco OR Mozambique OR Namibia OR Niger OR Nigeria OR Reunion OR Rwanda OR Senegal OR Seychelles OR ‘Sierra Leone’ OR Somalia OR ‘South Sudan’ OR Sudan OR Swaziland OR Tanzania OR Togo OR Tunisia OR Uganda OR ‘Western Sahara’ OR Zambia OR Zimbabwe)) AND ((MH ‘Mentally Disabled Persons’) OR (MH ‘Mental Disorders’) OR (MH ‘Psychotic Disorders+’) OR (MH ‘Schizophrenia+’) OR (MH ‘Bipolar Disorder+’) OR TI (‘mentally ill’ OR ‘severe mental’ OR psychoses OR psychosis OR psychotic OR schizo* OR bipolar OR ‘psychiatric disabilit*’ OR ‘psychosocial disabilit*’ OR ‘psycho-social disabilit*’ OR ‘psychiatric disable*’ OR ‘psychosocial disable*’ OR ‘major depress*’) OR AB (‘mentally ill’ OR ‘severe mental’ OR psychoses OR psychosis OR psychotic OR schizo* OR bipolar OR ‘psychiatric disabilit*’ OR ‘psychosocial disabilit*’ OR ‘psycho-social disabilit*’ OR ‘psychiatric disable*’ OR ‘psychosocial disable*’ OR ‘major depress*’)) AND ((MH ‘Absenteeism’) OR (MH ‘Recovery’) OR (MH ‘Sick Leave’) OR (MH ‘Disability Evaluation+’) OR (MH ‘Rehabilitation, Vocational+’) OR (MH ‘Sickness Impact Profile’) OR (MH ‘Occupational Health+’) OR (MH ‘Job Re-Entry’) OR TI (‘return to work’ OR (evaluation* AND (disability OR ‘work capacity’)) OR ‘work disability’ OR ‘work incapacity’ OR ‘work incapability’ OR ‘work inhibition’ OR ‘working incapacity’ OR ‘medical leave’ OR ‘sick leave’ OR ‘disability leave’ OR absente* OR ‘work absence’ OR ‘disability absence’ OR convalescen* OR ‘sick day*’ OR ‘illness day*’ OR ‘recovery of function’ OR ‘functional recovery’ OR (recovery AND function*) OR reintegration OR reemployment OR ‘job reentry’ OR presenteeism OR ‘sickness absence’ OR ‘work absenteeism’ OR ‘work day loss’ OR ‘work time loss’ OR ‘work productivity’ OR ‘work function*’ OR ‘work participation’ OR ‘work performance’ OR ‘performance at work’ OR ‘employment status’ OR ‘work status’ OR ‘occupational health’ OR employment OR labor OR work OR working OR workplace* OR occupation* OR vocation* OR ‘sheltered work*’) OR AB (‘return to work’ OR (evaluation* AND (disability OR ‘work capacity’)) OR ‘work disability’ OR ‘work incapacity’ OR ‘work incapability’ OR ‘work inhibition’ OR ‘working incapacity’ OR ‘medical leave’ OR ‘sick leave’ OR ‘disability leave’ OR absente* OR ‘work absence’ OR ‘disability absence’ OR convalescen* OR ‘sick day*’ OR ‘illness day*’ OR ‘recovery of function’ OR ‘functional recovery’ OR reintegration OR reemployment OR ‘job reentry’ OR presenteeism OR ‘sickness absence’ OR ‘work absenteeism’ OR ‘work day loss’ OR ‘work time loss’ OR ‘work productivity’ OR ‘work function*’ OR ‘work participation’ OR ‘work performance’ OR ‘performance at work’ OR ‘employment status’ OR ‘work status’ OR ‘occupational health’ OR employment OR labor OR work OR working OR workplace* OR occupation* OR vocation* OR ‘sheltered work*’) )
